# Quality of Life in Slovak Breast Cancer Survivors: A Cross-Sectional Study Using EORTC QLQ-C30 and BR23

**DOI:** 10.3390/nursrep16050162

**Published:** 2026-05-11

**Authors:** Petra Zuborova, Alica Slamkova, Milos Mlyncek, Jozef Visnovsky, Jan Bujnak, Pavol Zubor

**Affiliations:** 1 OBGY Health & Care Ltd., 01001 Zilina, Slovakia; zuborovap@gmail.com; 2 VISNOVSKI Ltd., 03601 Martin, Slovakia; jozo.visnovsky@gmail.com; 3 Department of Nursing, Faculty of Social Sciences and Health Care, Constantine the Philosopher University, 94901 Nitra, Slovakia; aslamkova@ukf.sk (A.S.); mlyncekmilos@hotmail.com (M.M.); 4 Department of Obstetrics and Gynaecology, Trencin Faculty Hospital, 91101 Trencin, Slovakia; 5 Department of Obstetrics and Gynaecology, Faculty of Medicine, Pavol Jozef Safarik University Hospital AGEL Kosice-Saca, Saca, 04015 Kosice, Slovakia; janbujnak@hotmail.com; 6 Department of Obstetrics and Gynaecology, Akershus University Hospital, 2212 Kongsvinger, Norway

**Keywords:** quality of life, breast cancer, patients, Slovakia, EORTC analysis, nursing

## Abstract

**Objectives:** This study sought to assess quality of life (QoL) in Slovak breast cancer survivors and examine its association with treatment modalities and sociodemographic factors. **Methods:** This cross-sectional observational study included 244 Slovak female breast cancer survivors (stages IA–IIIC). Participants were aged 25–85 years, ECOG 1–2, without synchronous malignancies or severe comorbidities. Data were collected using the EORTC QLQ-C30 and breast cancer-specific module. **Results:** Mean global health status/QoL indicated a moderate QoL level (51.67). Emotional and cognitive functioning were relatively preserved, whereas social and role functioning were more impaired. The most prominent symptoms included insomnia, fatigue, dyspnoea, and pain. Breast cancer-specific domains showed relatively high body image scores but marked impairment in sexual activity and future perspective. Severe symptoms included hair loss, upper limb problems and systemic side effects of therapy. Significant differences were observed across functional domains and symptoms (*p* < 0.001). Chemotherapy was associated with better role functioning (*p* = 0.011), lower pain (*p* = 0.043) and insomnia (*p* = 0.012), but also with higher levels of hair loss (*p* = 0.003); however, these findings should be interpreted with caution due to potential confounding factors. Radiotherapy was associated with higher social functioning, body image and future perspective (*p* = 0.007 for all), but also with increased breast symptom severity (*p* = 0.044). Sociodemographic factors, particularly place of residence and education level, were significantly associated with overall QoL. **Conclusions:** Slovak breast cancer survivors report moderate overall QoL, with emotional and cognitive functioning relatively preserved, but social, role and sexual domains impaired. Treatment modalities and sociodemographic factors are associated with differences in specific QoL domains, highlighting the need for targeted, context-sensitive supportive care. The findings also underscore the importance of oncology nursing in survivorship care, particularly in addressing physical, social and sexual domains and key symptoms such as insomnia, fatigue and pain. Targeted psychosocial and educational support, along with culturally sensitive QoL assessment, may help improve patient-centred outcomes in breast cancer survivors in Central and Eastern Europe.

## 1. Introduction

Breast cancer is among the most common malignant tumours affecting women worldwide, with approximately 2.3 million new cases diagnosed and 670,000 deaths recorded in 2022 [1]. It represents a major public health challenge due to its high incidence and severity, and both incidence and mortality are expected to rise moderately in the coming years. These trends are also reflected in Slovakia, where approximately 3300 new cases and over 800 breast cancer-related deaths are reported annually. In 2020, the age-standardized incidence rate in the Slovak Republic reached 109 cases per 100,000 women, exceeding the EU average [2,3]. Despite a relatively high Human Development Index (HDI) of 0.855, breast cancer mortality remains higher in Slovakia compared to countries with the highest HDI levels, likely due to limited access to modern therapies, insufficient follow-up care and differences in healthcare organization. Addressing these disparities is essential not only for reducing mortality but also for improving treatment outcomes, including quality of life (QoL), in breast cancer patients in Slovakia.

Globally, the burden of breast cancer is projected to increase substantially in the coming decade. Sha et al. [4] report that both incidence and mortality will rise by 2030 due to population ageing, lifestyle changes and improved detection. In the USA, approximately 4.3 million women were living with a history of invasive breast cancer as of 1 January 2025, with projections reaching 5.3 million by 2035 [5]. This growing prevalence underscores the need not only for effective treatment strategies but also for a comprehensive understanding of their impact on QoL. Survivorship extends beyond survival and includes symptom burden, psychosocial well-being, sexual health and functional capacity, all of which should be integrated into routine oncology care.

As the number of survivors increases, there is a growing demand for comprehensive QoL management. Care extends beyond medical treatment to include physical, psychological, sexual and social dimensions. Multidisciplinary teams are now standard in many countries, with oncology nurses playing an increasingly important role in collaboration with physicians, psychologists and rehabilitation specialists. The use of oncotelemedicine is also expanding to support continuity of care [6,7]. Supportive care programmes, such as the “Supportive Care Framework for Cancer Care” proposed by Fitch [8], have become established tools guiding the identification of patient needs and planning of care delivery, and are being incorporated into clinical guidelines, including the 2023 NCCN recommendations. These frameworks facilitate systematic identification and management of patient needs, thereby improving QoL.

QoL is influenced not only by treatment modalities—including surgery, chemotherapy, radiotherapy, immunotherapy and biological therapy—but also by multiple post-treatment factors such as lifestyle, rehabilitation, sexual health and psychological well-being. Treatment-related adverse effects significantly affect survivors’ daily functioning; these can include cognitive impairment, neuropathy, hormonal changes and local complications such as pain, lymphoedema and post-radiation effects [9]. Consequently, modern oncology care is shifting towards a patient-centred model emphasizing active patient involvement and holistic management, including psychosocial support.

Standardized instruments such as the EORTC QLQ-C30 and SF-36 are essential tools for assessing QoL in breast cancer patients. They enable objective evaluation of health status across multiple domains and support the planning of individualized follow-up care [10,11]. In advanced healthcare systems, QoL assessment is routinely integrated into clinical practice, and published data contribute to meta-analyses that inform clinical and nursing standards [12,13,14].

In Slovakia, the implementation of patient-centred care requires systematic QoL assessment across oncology centres at both the institutional and national levels. This is essential for developing targeted interventions to improve patient outcomes. To initiate this process in Slovakia, a detailed analysis of the current status of QoL surveillance and the consequences of treatment is needed. Given the limited availability of such data in Slovakia, our study sought to provide a comprehensive evaluation of QoL in breast cancer patients using standardized methodologies.

The objective of this study was thus to comprehensively evaluate the QoL of breast cancer patients following surgical and adjuvant treatment as a first detailed report from this region. The study sought to describe QoL scale scores in Slovak women during follow-up after treatment and to compare these findings with available international data, which remain limited. The study also examined differences across functional domains and symptom severity, highlighting potential targets for nursing and supportive care interventions; explored associations between treatment modalities and QoL outcomes; and evaluated the impact of selected sociodemographic factors on QoL perception.

## 2. Materials and Methods

### 2.1. Study Sample

A cross-sectional observational study was conducted. The analysed sample comprised *n* = 244 (100%) female respondents. Initially, 265 subjects were approached. Of these, 9 declined to participate, and 12 questionnaires were excluded due to incomplete responses or failure to meet inclusion criteria. Missing data at the item level were handled in accordance with the EORTC scoring manual, and only valid responses were included in the analyses. Participants were recruited in person by trained healthcare staff (oncological nurse) using a convenience sampling approach. Specifically, eligible patients were approached consecutively during their routine follow-up visits at oncogynaecological outpatient clinics. After being examined by their treating oncogynaecologist, patients who met the predefined inclusion criteria were invited to participate in the study and complete the questionnaire voluntarily. The EORTC QLQ-C30 questionnaire, along with the corresponding breast cancer-specific module, was administered anonymously, and patients completed it independently over the period of one year (March 2024–April 2025).

### 2.2. Participants and Data Collection

Participants were recruited from four oncology–gynaecology outpatient clinics in Slovakia (in the cities of Zilina, Banska Bystrica and Kosice). Patients were recruited using a convenience sampling approach, based on their availability and willingness to participate during routine follow-up visits. No randomization or consecutive sampling strategy was applied. Eligible patients were aged 25–85 years, 6–60 months post-completion of oncological treatment (surgery with or without adjuvant chemotherapy, radiotherapy, biological or immunotherapy); had histologically confirmed breast cancer, an Eastern Cooperative Oncology Group (ECOG) performance status of 1–2, and no synchronous malignancies; and provided written informed consent. Exclusion criteria included concurrent malignancies, ECOG status 3–5, significant chronic comorbidities (cardiovascular, endocrine, urological-nephrological, respiratory, immunological, or musculoskeletal), severe disability, and cognitive impairment or communication disorders.

The study was conducted in accordance with the ethical principles of the Declaration of Helsinki and was approved by the Ethics Committee (Institutional Review Board) of Constantine the Philosopher University in Nitra (approval ID: 316198). All participants were informed about the purpose of the study and provided written informed consent prior to participation. Data were collected anonymously and processed in accordance with applicable data protection regulations to ensure confidentiality.

### 2.3. Data Collection Instruments (Questionnaires)

The study employed two data collection instruments. The first was the European Organization for Research and Treatment of Cancer Quality of Life Questionnaire Core 30 (EORTC QLQ-C30, version 3.0). This standardized tool assesses QoL in cancer patients using a modular approach. It consists of a core questionnaire combined with specific modules tailored to cancer subgroups, allowing for both general and disease-specific evaluations. The core questionnaire includes 30 items covering five functional scales (physical, emotional, cognitive, social and role functioning); three symptom scales (fatigue, nausea/vomiting and pain); a global health status/QoL scale; and six single-item symptom measures (dyspnoea, insomnia, appetite loss, constipation, diarrhoea and financial difficulties). Most items are scored on a 4-point Likert scale ranging from 1 (not at all) to 4 (very much), while the global health status is rated on a 7-point scale from 1 (very poor) to 7 (excellent). According to the EORTC scoring manual, higher scores on functional scales and global health status indicate better functioning and higher QoL, whereas higher scores on symptom scales and single items indicate greater symptom severity and poorer patient condition [15].

The second instrument was the EORTC QLQ-BR23 module for breast cancer patients. This module includes four functional scales assessing body image, future perspective, sexual activity and sexual satisfaction, as well as four symptom scales evaluating systemic therapy side effects, hair loss distress, upper limb problems and breast-related issues. Scoring follows the same methodology as the functional and symptom scales of the QLQ-C30 questionnaire, with all scales and individual items ranging from 0 to 100. Higher scores on functional scales indicate better (healthier) functioning, whereas higher scores on symptom scales denote greater symptom burden. Items 44, 45 and 46 were reverse-coded prior to analysis (1 → 4, 2 → 3, 3 → 2, 4 → 1) [16]. For both questionnaires, results were analysed according to the official scoring manuals, and findings are presented in tables and graphs in the Section 3.

The internal consistency of the questionnaire scales was assessed using Cronbach’s alpha coefficient in the study sample.

### 2.4. Statistical Analysis

Statistical analyses were performed using MedCalc^®^ v23.3.7, MS Excel and IBM SPSS Statistics for Windows, version 31.0 (IBM Corp., Armonk, NY, USA). MedCalc^®^ and SPSS were used complementarily: MedCalc^®^ was primarily applied for descriptive statistics and non-parametric analyses, whereas SPSS was used for regression analyses and data management. Descriptive statistics were calculated for all variables. Data normality was assessed using the Shapiro–Wilk test. Between-group differences were analysed with the Mann–Whitney U test, and repeated measures were evaluated using Friedman’s ANOVA for within-subject comparison of QoL domains. Multiple linear regression was conducted to explore associations between selected variables and overall QoL. Given the exploratory nature of the analysis, the regression results are presented primarily to identify potential associations rather than to provide definitive predictive or causal inference. All variables included in the regression model were selected based on clinical relevance and availability within the dataset. Categorical variables (place of residence, chemotherapy, radiotherapy) were entered as binary indicators. Given the exploratory nature of the study and the sample size, no interaction terms were included. Model assumptions were assessed using standard diagnostic procedures. Statistical significance was set at α = 0.05, with *p* ≤ 0.05 considered statistically significant. The internal consistency of the EORTC QLQ-C30 and QLQ-BR23 scales was evaluated using Cronbach’s alpha coefficients calculated for each multi-item scale in the study sample. Values of ≥0.70 were considered indicative of acceptable reliability. Due to the exploratory nature of the study and the analysis of multiple QoL domains, no formal correction for multiple comparisons (e.g., Bonferroni or false discovery rate) was applied. Such corrections may increase the risk of type II error and obscure potentially clinically relevant findings. The results should therefore be interpreted with caution, considering the potential for type I error. The graphical plots are used for exploratory visualization of group differences. The fitted regression lines are presented for illustrative purposes only and do not imply a true linear relationship between variables, particularly in the case of binary predictors.

## 3. Results

The socio-demographic and clinical characteristics of breast cancer patients were as follows: The study group consisted of patients with a mean age of 57 years (standard deviation [SD] 16 years), ranging from 32 to 84 years, and a median age of 55 years. Regarding educational background, most patients had completed secondary education with a school-leaving certificate (60%), 35% held a university degree and 5% had undergone secondary education without a school-leaving certificate; none had only primary education. Concerning place of residence, 55% lived in rural areas, while 45% were from urban settings. In terms of treatment modality, 91.1% of patients underwent surgical intervention. Regarding adjuvant therapy, 31.6% received chemotherapy, 42.6% underwent radiotherapy alone, and 20.1% were treated with a combination of chemotherapy and radiotherapy. Patients who received neither chemotherapy nor radiotherapy and were treated solely by surgery accounted for 5.7% of the sample. Disease stages ranged from IA to IIIC accordingly pTNM staging system [17].

### 3.1. Evaluation of the EORTC QLQ-C30 and EORTC QLQ-BR23 Questionnaires in Slovak Breast Cancer Patients

The internal consistency of the EORTC QLQ-C30 scales was assessed using Cronbach’s alpha coefficients (Table 1). Overall, the questionnaire demonstrated acceptable to excellent internal consistency across the evaluated multi-item scales. Most functional and symptom scales showed good reliability. Lower internal consistency was observed for the nausea and vomiting scale, which should therefore be interpreted with caution.

Following the assessment of internal consistency, Table 2 presents the mean score values reflecting the functional status of the respondents in relation to the overall health/QoL item, five functional scales, three symptom scales, and six individual items of the EORTC QLQ-C30 questionnaire and the BR23 module. In accordance with the EORTC scoring system, higher scores on functional scales represent better functioning, whereas higher scores on symptom scales indicate greater symptom burden.

The results indicate that the mean score for subjective overall health and QoL was 51.67, which reflects a moderate perceived health status. Physical functioning showed slightly above-average levels, which suggests a relative ability to perform daily activities despite some limitations. Role functioning was lower, indicating restrictions in daily responsibilities such as work, family and social activities. Emotional functioning remained relatively high, although with some variability among patients, while cognitive functioning showed the highest scores, suggesting preserved concentration and memory.

In contrast, social functioning demonstrated the lowest scores, with notable variability and limitations in social interactions. The most prominent symptoms included insomnia, fatigue, dyspnoea and pain, whereas other symptoms such as diarrhoea, vomiting, constipation and appetite loss, as well as financial impact, were less pronounced. Overall, patients maintained relatively good emotional and cognitive functioning, while social and role domains were the most affected.

Similar reliability patterns were observed for the disease-specific QLQ-BR23 module, supporting the consistency of both instruments in this study. The internal consistency of the EORTC QLQ-BR23 scales showed a similar pattern (Table 3). Overall, the BR23 module demonstrated acceptable to excellent internal consistency across the evaluated multi-item scales. Lower internal consistency was observed for the systemic side effects scale, which should be interpreted with caution.

The EORTC QLQ-BR23 module was used to assess selected domains related to functional status and symptom burden in patients with breast cancer (see Table 4).

Participants scored highest on the body image scale (mean 71.67), suggesting that this domain was relatively less impaired, although variability in the scores suggests individual differences in body perception. In contrast, future perspective scored low (mean 30.00), reflecting a generally less optimistic outlook. Sexual activity was notably impaired (mean 13.32), while sexual satisfaction averaged 46.15 based on responses from 186 participants, which may limit the reliability of this finding.

Overall, sexual activity, future perspective and sexual satisfaction were the most affected domains, likely reflecting the physical, emotional and social burden associated with the disease and its treatment. The most prominent symptoms included hair loss, upper limb problems and systemic therapy side effects, all of which may interfere with daily functioning, whereas breast symptoms were less severe.

### 3.2. Differences in QoL Across Functional Scales and Symptom Severity: Potential Targets for Nursing Interventions

This study also sought to determine whether differences exist in QoL across functional scales and to establish their relative severity, thus identifying priority areas for nursing interventions. The Friedman test was used to compare QoL levels across dependent samples, while scales with insufficient responses (e.g., sexual satisfaction) were excluded from the analysis.

Significant differences in QoL across functional scales were observed (*p* < 0.001). The highest scores were found in cognitive functioning (M = 81.67) and emotional functioning (M = 74.58), followed by body image (M = 71.67) and physical functioning (M = 67.33). More pronounced declines were observed in role functioning (M = 54.17) and social functioning (M = 49.17), while the lowest scores were reported in sexual functioning (M = 13.32) and future perspective (M = 30.00) (see Table 5).

The analysis of symptom severity among breast cancer patients revealed significant differences between symptom groups (χ^2^(11) = 103.311, *p* < 0.001). The most severe symptoms included insomnia (M = 40.00), fatigue (M = 36.11), dyspnoea (M = 33.33), pain (M = 32.50) and appetite loss (M = 23.33). These were followed by systemic therapy side effects (M = 23.33), upper limb problems (M = 23.89) and constipation (M = 20.00). In contrast, the least severe symptoms were diarrhoea (M = 8.33), nausea and vomiting (M = 8.33), financial difficulties (M = 16.67), and breast-related symptoms (M = 18.75) (see Table 6).

### 3.3. Examination of the Association Between Treatment Modalities (Chemotherapy, Radiotherapy) and QoL Outcomes Across Functional Domains/Scales and Symptoms

We applied the Mann–Whitney U test to examine whether differences in QoL were present according to the type of treatment received. This non-parametric test compared QoL functional domains and symptom severity between patients who underwent chemotherapy or radiotherapy and those who did not. The association with surgical treatment was not analysed due to its near-universal application among the cohort (91.1%).

#### 3.3.1. Association of Chemotherapy with Functional Domains in Breast Cancer Patients

**Figure 1:**
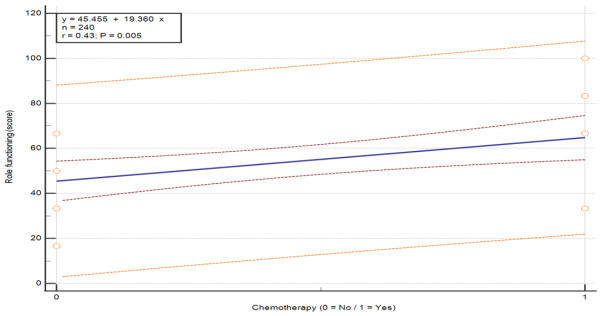
Relationship between QoL scores in the functional scale “Role functioning” and receipt of chemotherapy in patients with breast cancer. Values are presented on a 0–100 scale according to the EORTC scoring manual, where higher scores indicate better functioning. The plot illustrates the distribution of individual patient scores according to chemotherapy status (no/yes). The central line represents the fitted linear regression model (y = a + bx). The inner red dashed lines represent the 95% confidence interval of the regression line, and the outer orange dashed lines represent the 95% prediction interval. The horizontal axis represents treatment group, and the vertical axis represents QoL score. Abbreviations: QoL—quality of life.

The analysis of chemotherapy revealed a statistically significant difference in only one QoL domain—role functioning, U = 108.0, *p* = 0.011 (see Table 7). Patients who did not receive chemotherapy reported significantly lower scores in role functioning (M = 45.46, SD = 16.41) compared to those who underwent chemotherapy (M = 64.82, SD = 24.85). This difference in role functioning between patients who did and did not receive chemotherapy is illustrated in Figure 1 (*p* = 0.005).

#### 3.3.2. Association of Chemotherapy with Symptom Severity in Breast Cancer Patients

**Figure 2:**
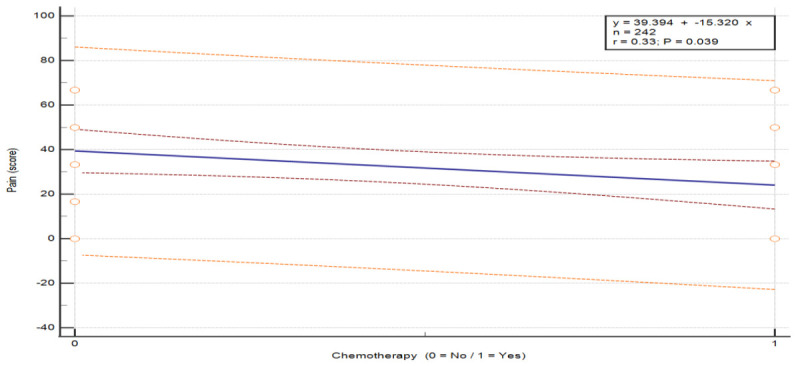
The relationship between the severity of the symptom “Pain” according to chemotherapy status in patients and breast cancer. Values are presented on a 0–100 scale according to the EORTC scoring system, where higher scores indicate greater symptom severity. The plots illustrate the distribution of individual patient responses according to chemotherapy status (no/yes). The central line represents the fitted linear regression model (y = a + bx). The inner red dashed lines represent the 95% confidence interval of the regression line, and the outer orange dashed lines represent the 95% prediction interval. The horizontal axis represents treatment group, and the vertical axis represents symptom score. Abbreviations: QoL—quality of life.

**Figure 3:**
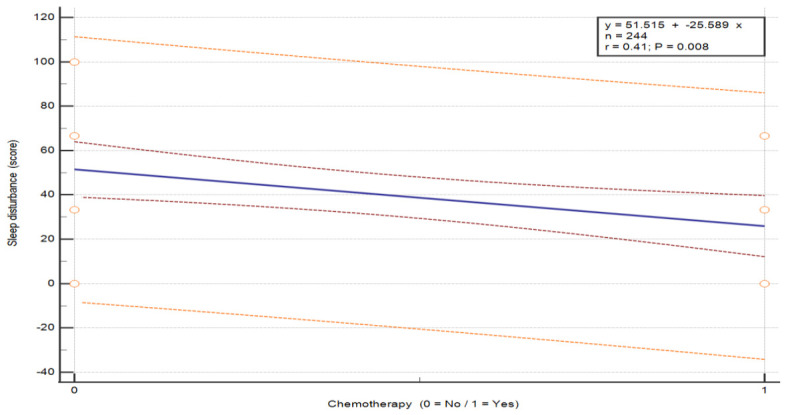
The relationship between the severity of the symptom “Sleep disturbance” according to chemotherapy status in patients with breast cancer. Values are presented on a 0–100 scale according to the EORTC scoring system, where higher scores indicate greater symptom severity. The plots illustrate the distribution of individual patient responses according to chemotherapy status (no/yes). The central line represents the fitted linear regression model (y = a + bx). The inner red dashed lines represent the 95% confidence interval of the regression line, and the outer orange dashed lines represent the 95% prediction interval. The horizontal axis represents treatment group, and the vertical axis represents symptom score. Abbreviations: QoL—quality of life.

**Figure 4:**
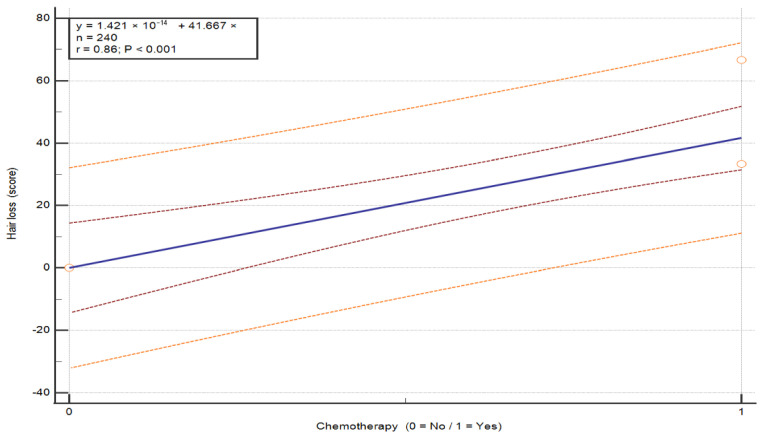
The the severity of the symptom “Hair loss” according to chemotherapy status in patients with breast cancer. Values are presented on a 0–100 scale according to the EORTC scoring system, where higher scores indicate greater symptom severity. The plots illustrate the distribution of individual patient responses according to chemotherapy status (no/yes). The central line represents the fitted linear regression model (y = a + bx). The inner red dashed lines represent the 95% confidence interval of the regression line, and the outer orange dashed lines represent the 95% prediction interval. The horizontal axis represents treatment group, and the vertical axis represents symptom score. Abbreviations: QoL—quality of life.

The analysis of the association between chemotherapy and symptom severity in breast cancer patients revealed statistically significant differences in three symptom categories: pain (U = 130.0, *p* = 0.043), insomnia (U = 110.0, *p* = 0.012) and hair loss (U = 0, *p* = 0.003) (see Table 8). Patients who did not undergo chemotherapy reported higher pain (M = 39.39 vs. 24.07) and insomnia (M = 51.52 vs. 25.93) compared to those who received chemotherapy. In contrast, hair loss was markedly higher in patients after chemotherapy (M = 41.67) and absent in those without chemotherapy (M = 0). These findings indicate that patients without chemotherapy experienced greater pain and insomnia, whereas chemotherapy was associated with increased hair loss. These differences are visually depicted in the exploratory regression plots (Figure 2: pain, *p* = 0.039; Figure 3: insomnia, *p* = 0.008; Figure 4: hair loss, *p* = 0.001).

#### 3.3.3. Association of Radiotherapy with Functional Domains in Breast Cancer Patients

**Figure 5:**
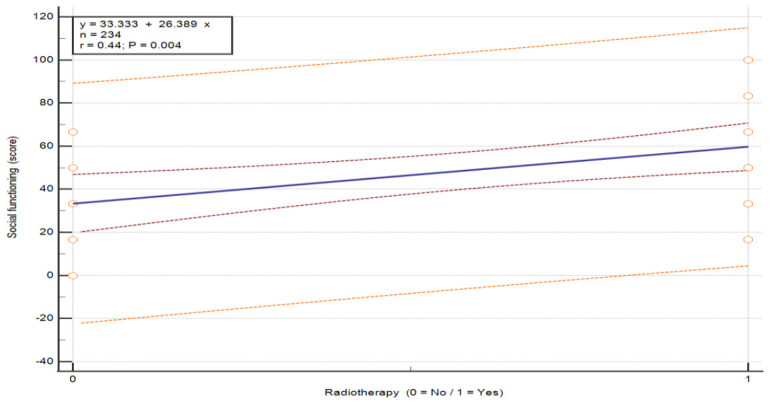
Association between QoL in the scale “Social functioning” and radiotherapy status in patients with breast cancer. Values are presented on a 0–100 scale according to the EORTC scoring manual, where higher scores indicate better functioning. The plots show the distribution of QoL scores according to radiotherapy status (no/yes). The central line represents the fitted linear regression model (y = a + bx), with the inner red dashed lines represent the 95% confidence interval of the regression line, and the outer orange dashed lines represent the 95% prediction interval. The horizontal axis represents treatment group, and the vertical axis represents QoL score. Abbreviations: QoL—quality of life.

**Figure 6:**
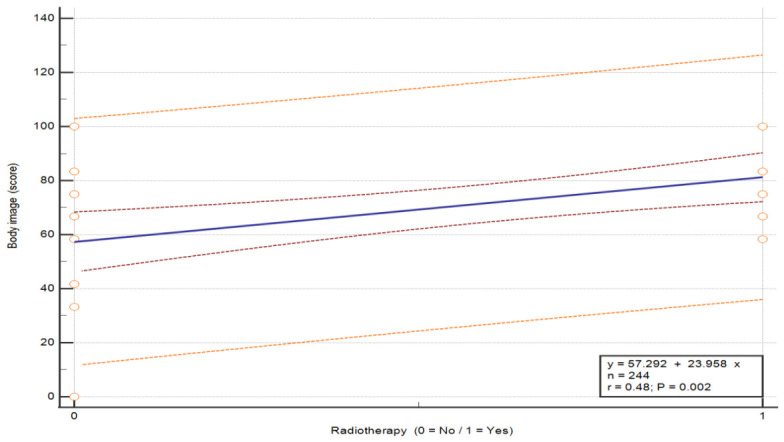
Association between QoL in the scale “Body image” and radiotherapy status in patients with breast cancer. Values are presented on a 0–100 scale according to the EORTC scoring manual, where higher scores indicate better functioning. The plots show the distribution of QoL scores according to radiotherapy status (no/yes). The central line represents the fitted linear regression model (y = a + bx), with the inner red dashed lines represent the 95% confidence interval of the regression line, and the outer orange dashed lines represent the 95% prediction interval. The horizontal axis represents treatment group, and the vertical axis represents QoL score. Abbreviations: QoL—quality of life.

**Figure 7:**
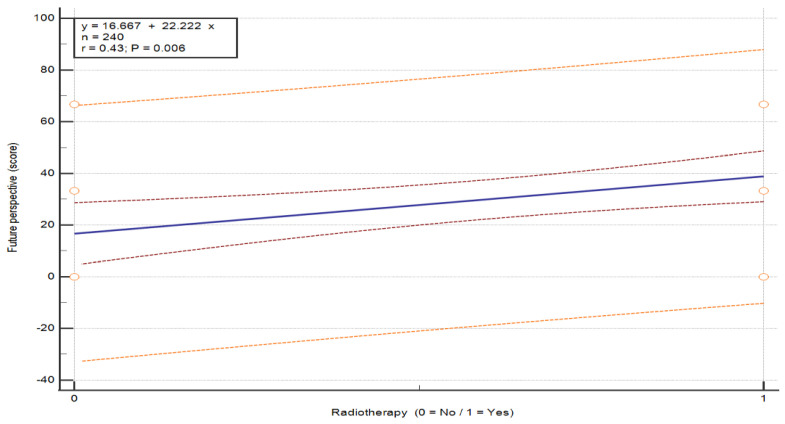
Association between QoL in the scale “Future perspective” and radiotherapy status in patients with breast cancer. Values are presented on a 0–100 scale according to the EORTC scoring manual, where higher scores indicate better functioning. The plots show the distribution of QoL scores according to radiotherapy status (no/yes). The central line represents the fitted linear regression model (y = a + bx), with the inner red dashed lines represent the 95% confidence interval of the regression line, and the outer orange dashed lines represent the 95% prediction interval. The horizontal axis represents treatment group, and the vertical axis represents QoL score. Abbreviations: QoL—quality of life.

The analysis of the association between radiotherapy and QoL in breast cancer patients, performed using the Mann–Whitney U test, revealed statistically significant differences in three functional scales: social functioning (U = 96.0, *p* = 0.007), body image (U = 96.0, *p* = 0.007) and future perspective (U = 100.0, *p* = 0.007) (see Table 9). Patients who did not undergo radiotherapy reported lower QoL in social functioning (M = 33.33 vs. 59.72), body image (M = 50.00 vs. 52.78) and future perspective (M = 57.29 vs. 81.25) compared to those who received radiotherapy. Overall, radiotherapy was associated with higher QoL in these domains. These between-group differences are illustrated in Figure 5, Figure 6 and Figure 7 (*p* = 0.015, *p* = 0.007 and *p* = 0.027, respectively).

#### 3.3.4. Association of Radiotherapy with Symptom Severity in Breast Cancer Patients

**Figure 8:**
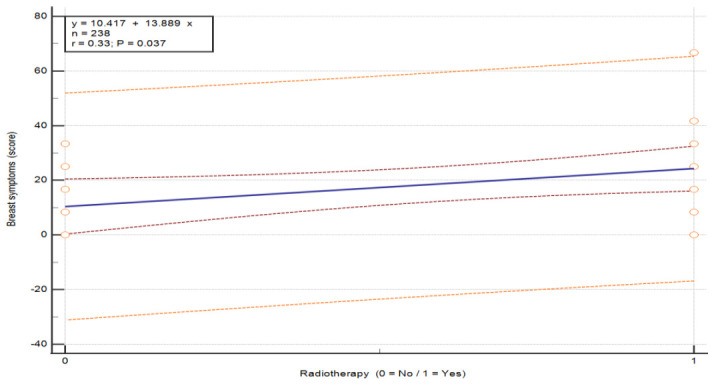
Association between “Breast symptom” severity and radiotherapy status in patients with breast cancer. Values are presented on a 0–100 scale according to the EORTC scoring system, where higher scores indicate greater symptom burden. The plot illustrates the distribution of breast-related symptom scores according to radiotherapy status (no/yes). The central line represents the fitted linear regression model (y = a + bx), with the inner red dashed lines represent the 95% confidence interval of the regression line, and the outer orange dashed lines represent the 95% prediction interval. The horizontal axis represents treatment group, and the vertical axis represents symptom score. Abbreviations: QoL—quality of life.

In the case of radiotherapy, a statistically significant difference was observed in only one symptom group (breast symptoms) between patients who had undergone this treatment and those who had not (U = 124.0, *p* = 0.044; see Table 10). Patients without radiotherapy reported lower severity of breast-related problems (M = 10.42) compared to those who received radiotherapy (M = 24.31). This higher symptom severity in the radiotherapy group is further illustrated by the exploratory regression plot in Figure 8 (*p* = 0.036).

### 3.4. Evaluation of the Association Between Sociodemographic Factors and Individual QoL Perception

We then conducted a multiple linear regression analysis to investigate which factors were significantly associated with overall health and QoL in patients with breast cancer diseases. In the regression model, the dependent variable was the level of overall health and QoL, while independent variables included patient age (a non-modifiable factor), education level, place of residence and treatment modalities—chemotherapy and radiotherapy (modifiable factors). Surgical treatment was excluded from the model, as it was undergone by most of the patients (91.1%). Age and education were measured on an interval scale, while place of residence (urban vs. rural), chemotherapy (no/yes) and radiotherapy (no/yes) were measured on a nominal scale.

**Figure 9:**
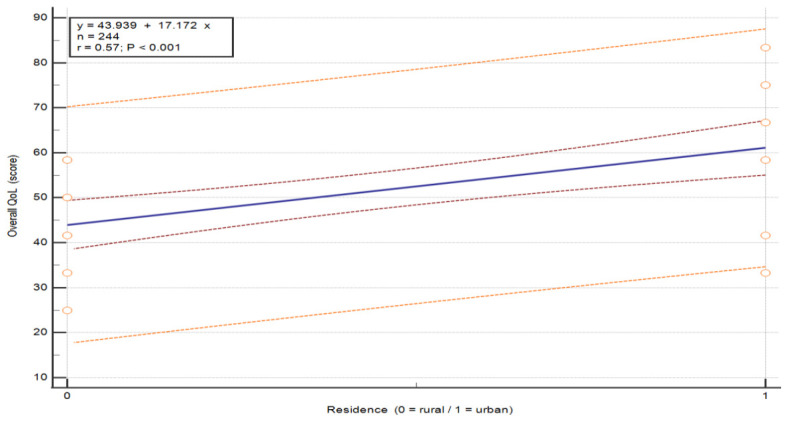
Association between overall QoL and place of residence in patients with breast cancer. The plots illustrate the relationship between overall QoL score and selected independent variables. Each point represents an individual patient. The central line represents the fitted linear regression model (y = a + bx). The inner red dashed lines represent the 95% confidence interval of the regression line, and the outer orange dashed lines represent the 95% prediction interval. These analyses are exploratory and do not imply causality. Abbreviations: QoL—quality of life.

**Figure 10:**
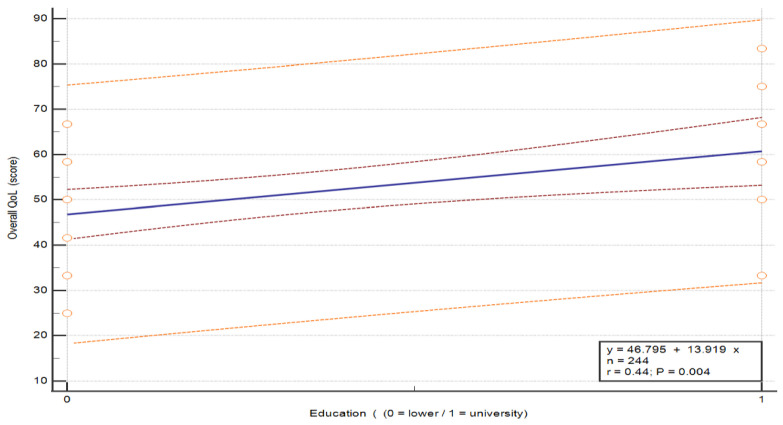
Association between overall QoL and level of education in patients with breast cancer. The plots illustrate the relationship between overall QoL score and selected independent variables. Each point represents an individual patient. The central line represents the fitted linear regression model (y = a + bx). The inner red dashed lines represent the 95% confidence interval of the regression line, and the outer orange dashed lines represent the 95% prediction interval. These analyses are exploratory and do not imply causality. Abbreviations: QoL—quality of life.

Analysis of the breast cancer patient cohort revealed that overall health and QoL were significantly associated with place of residence, with a regression coefficient of B = −13.402, *p* < 0.001. The negative direction of the coefficient indicates that patients living in urban areas had significantly higher overall health and QoL scores than those living in rural areas. Place of residence emerged as a statistically significant factor associated with overall health and QoL scores, with a moderate effect size (β = −0.443). While the overall model demonstrated moderate explanatory power (adjusted R^2^ = 0.253), place of residence remained one of the most influential variables within the model. Similarly, education level was also significantly associated with QoL with higher education corresponding to higher QoL scores, with a regression coefficient of B = −5.214, *p* = 0.018 (β = −0.193) (see Table 11 and regression-based visualization plots in Figure 9 and Figure 10).

**Figure 11:**
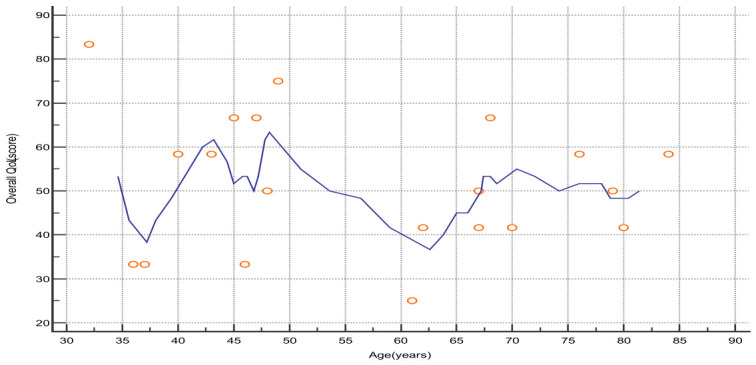
Level of overall QoL of breast cancer patients according to age distribution. The scatter plot with a moving average line illustrates the distribution of overall QoL scores across age groups. Each point represents an individual patient, and the smoothed line represents a moving average calculated over 5-year intervals. Observed patterns are descriptive and should be interpreted cautiously, as no statistically significant association is identified. Abbreviations: QoL—quality of life.

No statistically significant association was found when assessing the relationship between overall QoL and age in breast cancer patients using linear regression. However, exploratory descriptive analyses across age intervals suggested potential variability in QoL scores. Specifically, lower QoL levels were observed in patients aged 35–40 and 55–65 years (see Figure 11). These observations should be interpreted with caution, as they do not represent statistically significant differences and may reflect sample variability rather than true age-related effects. Nevertheless, these findings highlight the importance of considering age-group stratification when planning supportive care strategies for breast cancer patients.

## 4. Discussion

Oncogynaecological diseases are increasing worldwide and thus represent a growing focus in both treatment and preventive strategies, including nursing care [18,19,20]. Beyond recurrence surveillance, long-term follow-up increasingly emphasizes the maintenance of QoL, which requires continuous and structured interaction between patients and healthcare providers through a more involved nursing-based approach [21]. In this context, and given the limited availability of data from Slovakia, our study provides region-specific insight into QoL in breast cancer survivors, thus contributing to a better understanding of survivorship in this setting.

Globally, QoL in breast cancer patients has shown a gradual improvement over time. A large meta-analysis by Mokhtari-Hessari and Montazeri [22], covering 974 studies, demonstrated a positive trend, particularly in the last decade, and highlighted the role of relatively simple interventions, such as physical activity and psychosocial support. However, persistent challenges remain, particularly in symptom control (e.g., pain, lymphoedema), as well as in domains related to fear, sexual functioning and future perspective, especially among younger patients. Importantly, despite methodological improvements in QoL assessment, these findings suggest that certain patient-relevant dimensions remain insufficiently addressed. Recognizing that QoL is associated with treatment outcomes [23], its systematic assessment represents a critical component of modern oncology care rather than a supplementary measure.

Effective patient–provider communication represents a key mechanism through which QoL may be influenced. Adequate information regarding diagnosis, treatment modalities and expected outcomes has been associated with more favourable perceptions of the disease trajectory, lower decisional regret and better functioning across QoL domains [24]. In this context, communication may act not only as supportive care but also as a determinant of how patients interpret and adapt to their condition.

Breast cancer patients experience a complex spectrum of symptoms resulting from both the disease itself and its treatment, which interact and may differentially affect individual QoL domains. Although the overall QoL scores indicate a moderate level, impairments in specific domains such as social and role functioning suggest a clinically meaningful burden affecting daily activities and social reintegration. This is consistent with our findings, where most women reported physical problems that interfered with their normal lives. Respondents in our study coped relatively well with the emotional and cognitive demands associated with breast cancer. In contrast, social functioning and the ability to manage life roles were most affected, likely reflecting the cumulative impact of physical symptoms, treatment-related limitations and changes in social roles. This apparent discrepancy between the relative preservation of emotional functioning and impairments in other domains may be partially explained by adaptive coping strategies and response shift, where patients adjust their internal standards and expectations over time.

Insomnia, which showed the highest average score, suggests that sleep disturbances may represent an important underlying mechanism contributing to impairments in physical regeneration, emotional stability and overall health. These findings are supported by studies from Emre et al. [25], Hendy et al. [26] and Durán-Gómez et al. [27]. Collectively, these studies highlight the critical need to monitor and address sleep disturbances in breast cancer patients as an integral part of comprehensive care. Targeted support in this area may therefore be particularly relevant, as patients often do not report sleep-related difficulties or may not recognize their broader impact on daily functioning during and after treatment.

Overall, our findings are consistent with international studies, which similarly report that physical symptoms and psychosocial factors remain key determinants of QoL in breast cancer survivors. However, some differences may be attributable to variations in healthcare systems, access to supportive care and sociocultural factors across countries, particularly between Western Europe and Central and Eastern European regions. In particular, the prominence of sleep disturbances and social role limitations observed in our cohort is in line with findings from large international datasets, where these domains are consistently identified among the most affected aspects of survivorship. Moreover, the relative preservation of emotional and cognitive functioning in our sample may reflect adaptive coping mechanisms and ongoing follow-up care, as described in other European populations.

Similar conclusions were reported in a meta-analysis by Heidary et al. [12], which reviewed over 1565 publications addressing physical, spiritual and psychological dimensions of QoL in breast cancer patients. The authors differentiated between patients without recurrence and those with metastatic disease, identifying social relationships as a key determinant of QoL in non-metastatic patients, whereas concerns related to survival, future planning and worries about their children’s futures were more prominent in advanced disease. Likewise, Khajoei et al. [13] identified multiple domains of patient needs, with information provision, physical activity, daily functioning, interpersonal relationships, psychological and emotional support, and intimacy consistently emerging as priority areas. Taken together, these findings support the interpretation that QoL impairment is multidimensional and that the deficits observed in our cohort, particularly in social and role functioning, likely reflect broader unmet supportive care needs rather than isolated symptoms.

Assessing QoL in patients with malignant diseases is crucial, as it reflects not only disease burden but also the broader context of treatment-related and systemic factors, including access to therapies, healthcare organization and social support. QoL is generally higher in developed countries compared to regions with less developed healthcare systems, such as Central and Eastern Europe [28,29]. For example, a large international study by Nolte et al. [30], based on 15,386 respondents across multiple countries, demonstrated significant cross-country differences in QoL, as well as variability related to age and gender, highlighting the importance of contextual interpretation of QoL data. Given these disparities, direct comparison of our results with data from Western European or other high-resource settings should be approached with caution. Differences in treatment availability, follow-up care and supportive services may substantially influence QoL outcomes and limit the comparability of findings. Therefore, rather than focusing on absolute comparisons, our results should be interpreted in terms of patterns of impairment across QoL domains. In this context, the domains most affected in our cohort are consistent with international observations, thus supporting the external validity of our findings despite systemic differences.

At the national level, available data remain scarce. Only two publications, both from the same research group, were identified [31,32]. These studies assessed psychosocial support in breast cancer patients and revealed increased emotional stress and insufficient psychosocial support from healthcare providers and psychologists. Because we did not evaluate the type of surgery, direct comparison is not possible. Nevertheless, continued analyses and publications are important for improving patient communication and proposing effective interventions to enhance QoL. Such work may also gradually provide a domestic basis for comparison with international studies from countries with highly developed healthcare systems, including Scandinavian settings [33,34,35,36].

Although our study was retrospective and did not assess QoL longitudinally, the observed variability across age groups suggests that QoL is not static but may change over time. This underscores the importance of longitudinal and prospective assessment approaches, which may better capture the dynamic nature of survivorship and identify critical periods for intervention. We illustrated this concept using graphs depicting fluctuations in overall QoL across age intervals. In line with findings from the same regional area, long-term monitoring of QoL in gynaecological oncology patients appears to be essential [37]. A prior study demonstrated that the type of surgery is associated with differences in sexual satisfaction, body image and future perspective, thus underscoring the need for continuous, individualized assessment. Furthermore, the association between age and QoL was supported by Arthur et al. [14], who showed that limitations in physical health and social relationships were more pronounced in older patients, particularly those aged over 65 years. Collectively, these findings reinforce the need for age-adapted and individualized survivorship care strategies.

In our study, breast cancer patients frequently exhibited the lowest scores in the domains of body image, sexual function and emotional well-being. These impairments likely reflect the combined impact of treatment-related physical changes and their psychological consequences. Breast loss following mastectomy, alterations in body perception, scarring, limited upper limb mobility, lymphoedema and chemotherapy-induced alopecia represent key factors contributing to psychological distress and reduced self-perception.

Anxiety and depression may further exacerbate impairments in QoL. Similar findings have been reported in other studies. For example, a systematic review and meta-analysis by Ma et al. [38] demonstrated that telemedicine-based psychosocial interventions improved QoL and reduced anxiety and depression among breast cancer patients. Battistello et al. [39] found that body image perception and QoL in women after surgical treatment of breast cancer were significantly influenced by factors such as age, use of psychotropic medications and negative body image perception. Furthermore, Yao et al. [40] reported that higher levels of body image concerns and psychological stress were associated with lower QoL, particularly in younger breast cancer patients. These findings suggest that psychological distress may act both as a consequence of physical changes and as an independent factor influencing QoL outcomes.

In our cohort, another frequently affected domain was sexual intimacy, a finding consistent with the literature. Sexual life in breast cancer patients is often adversely affected and is associated with altered body image, loss of libido, hormonal changes and concerns regarding future life prospects. These observations are consistent with the findings of Franzoi et al. [41], who reported that 78.2% of patients experienced at least one sexual problem, including low sexual activity, negative body image and sexual dysfunction, all of which were associated with lower QoL. Similarly, Gan et al. [42] found that 81% of young breast cancer patients experienced sexual dysfunction, including reduced sexual interest, arousal difficulties, orgasmic problems and vaginal lubrication issues, which were associated with impairments in both sexual life and overall QoL. Tian et al. [43] prospectively demonstrated that sexual activity declined markedly during treatment, with only 39.1% of patients remaining sexually active one month post-diagnosis. Importantly, age, libido and vaginal lubrication were positively correlated with sexual activity, highlighting the multifactorial nature of sexual dysfunction in this population. Collectively, these findings, alongside our results, underscore the critical need for comprehensive care that addresses both the physical and psychological dimensions of treatment. Such care is important in supporting patients in maintaining and improving their overall QoL, with particular attention to body image, sexual health and psychosocial well-being.

In line with findings reported in international studies, the greatest decline in QoL in our cohort was observed in patients undergoing active treatment, particularly chemotherapy. These findings are consistent with the well-documented burden of treatment-related toxicity, where side effects such as fatigue, nausea, vomiting, polyneuropathy, mucositis and taste changes, together with psychosocial isolation and reduced work activity, contribute to a decline in QoL. Importantly, these factors do not act in isolation but interact, amplifying their overall impact on both physical and psychosocial domains. These observations align with studies by Binotto et al. [44], Hajj et al. [45], Kırca et al. [46], Pellegrini et al. [47] and Smith et al. [48], and they underscore the multifactorial nature of chemotherapy-related QoL impairment in breast cancer patients.

Interestingly, in our cohort, patients who received chemotherapy reported higher scores in role functioning than those who did not. This finding may appear counterintuitive and should therefore be interpreted with caution. One possible explanation is that patients who did not receive chemotherapy may have differed in important baseline characteristics, such as age, comorbidities or treatment selection factors, which were not fully captured in the present analysis and may have influenced their perceived ability to maintain daily roles. In addition, patients undergoing chemotherapy may have received more intensive follow-up care and supportive attention, which may have contributed to better perceived role functioning in some individuals. Given the cross-sectional design of the study, this observation should be understood as an association rather than a causal effect. Taken together, these findings highlight the complexity of interpreting QoL outcomes in cross-sectional analyses, where observed differences may reflect underlying patient selection, treatment indication or survivorship bias rather than true treatment effects. This underscores the importance of cautious interpretation and the need for longitudinal designs to better disentangle these relationships.

Conversely, following the completion of active treatment, most QoL domains tend to show gradual improvement; nonetheless, certain symptoms, such as lymphoedema or chronic pain, may persist over the long term. This aligns with the findings of Jørgensen et al. [49], who demonstrated that breast cancer-related lymphoedema is associated with lasting impairments not only in physical functioning, such as mobility limitations and discomfort in the affected limb, but also in psychosocial aspects. Notably, their study revealed that the mere presence of lymphoedema, rather than its clinical severity, is associated with substantial impairments in patients’ QoL. These findings underscore the critical need for targeted rehabilitation strategies, ongoing monitoring and supportive care programmes to mitigate the long-term physical and psychosocial consequences of lymphoedema in breast cancer survivors.

An important finding of our study, consistent with the literature, is the persistence of reduced scores in psychological and social domains even several years after completion of treatment. This suggests that, despite physical recovery, psychosocial adaptation may remain incomplete in a substantial proportion of survivors. Fear of recurrence, decreased sexual activity, changes in partner relationships and difficulties with work reintegration may contribute to this prolonged burden and limit full return to pre-diagnosis QoL. Similar observations have been reported in multiple studies. Zhu et al. [50] found that fear of cancer recurrence was significantly associated with survivors’ ability to return to work, while higher health literacy attenuated this relationship. Ban et al. [51] showed that fear of cancer progression negatively correlated with QoL, partially mediated by social support. Hsiao et al. [52] demonstrated that post-surgical changes in QoL were closely linked to depression and other psychological factors, thus highlighting the need for integrated psychosocial care. Tan et al. [53] further emphasized that cancer and its treatment are frequently associated with psychiatric comorbidity, disruptions in family and social relationships, and impaired sexual functioning, all of which may persist long after treatment. These findings underscore that long-term QoL impairment is not solely driven by physical sequelae but also by sustained psychosocial stressors, which reinforces the need for multidisciplinary survivorship care.

Our results further emphasize the crucial role of support from family, healthcare teams and patient communities, as multiple studies have shown that psychosocial interventions, educational programmes and rehabilitation activities are associated with better QoL and lower depressive symptom burden. In a large meta-analysis, Hwang et al. [54] reported that cognitive interventions, meditation and psychological education were associated with reduced negative emotions and improved QoL in breast cancer patients. Notably, such interventions do not always require in-person attendance; recent evidence indicates that telemedicine-based psychosocial interventions are associated with improvements in QoL and reductions in distress, anxiety, fatigue, sleep disturbances, sexual dysfunction and fear of cancer recurrence [38]. These findings suggest that accessible and flexible models of supportive care may play an important role in addressing persistent unmet needs in survivorship.

Consistent with our findings and the growing body of evidence, QoL assessment should be considered an integral component of comprehensive breast cancer care rather than a supplementary outcome. Systematic symptom screening, timely intervention and a coordinated multidisciplinary approach, including oncologists, surgeons, psychologists, physiotherapists, nutritionists and oncology nurses, are essential not only for optimizing survival outcomes but also for achieving sustained improvements in patient-centred outcomes across the survivorship trajectory.

Although our study provides the first comprehensive and cohesive assessment of QoL in Slovak breast cancer patients following surgical and adjuvant treatment—offering detailed insights into medical care, follow-up and nursing support, as well as potential inclusion in meta-analyses across Central and Eastern Europe—it also has several limitations. The main limitations include the relatively small sample size and the cross-sectional design. Accordingly, the findings should be interpreted with caution, as the cross-sectional nature of the study only allows the identification of associations and precludes causal or temporal inference. Moreover, the sample size limits the ability to assess longitudinal changes in QoL or its relationship with pTNM disease stage at the individual level. Nevertheless, our findings provide relevant data underscoring the need for systematic monitoring and ongoing support of QoL in this patient population.

Another limitation is the lack of adjustment for certain clinical confounders, such as detailed disease stage stratification, specific treatment combinations and other relevant clinical variables. Although major comorbidities were excluded through the inclusion criteria, residual confounding cannot be fully ruled out. Due to the sample size and cross-sectional design, more detailed multivariable adjustment was not feasible. Future studies with larger, prospectively collected datasets should incorporate more comprehensive adjustment for clinical and treatment-related factors. The regression analysis should therefore be interpreted as exploratory, aiming to identify potential associations rather than to provide definitive predictive modelling.

Furthermore, another limitation of this study is the heterogeneity of the sample with respect to time since completion of treatment (6–60 months). This defined post-treatment interval also excludes patients in the very early post-treatment phase as well as long-term survivors beyond five years, both of whom may experience different QoL trajectories. QoL is known to vary across different survivorship phases, and patients in the early post-treatment period may experience different challenges compared to long-term survivors. However, the inclusion of patients across this broader time interval was intentional and reflects real-world clinical practice, where patients at different stages of survivorship are managed within the same follow-up setting. This approach allows for a more comprehensive assessment of the QoL burden across the survivorship continuum. Nevertheless, future studies should incorporate stratified analyses based on time since treatment to better capture phase-specific patterns and support the development of time-specific, tailored supportive care interventions across the survivorship continuum.

The use of a convenience sampling approach may also limit the generalizability of the findings and introduce selection bias, as patients who agreed to participate may differ systematically from those who declined participation. Additionally, the reliance on self-reported questionnaires introduces the potential for response bias, including recall bias and subjective interpretation of symptoms and QoL domains. These methodological limitations should be considered when interpreting the results, particularly in relation to external validity.

The urgency of strengthening oncological healthcare systems, with a greater emphasis on QoL assessment, is further supported by the findings of Vrdoljak et al. [55]. Based on the recommendations of the SEEROG (Southeast Europe Research Oncology Group), their analysis highlighted persistent disparities between Western European and Central and Eastern European countries, where higher mortality rates despite comparable or lower incidence suggest differences in the effectiveness of oncological care. These findings underscore the importance of improving not only survival outcomes but also the quality of survivorship care in Central and Eastern Europe. The SEEROG panel proposed several measures to address these gaps, including strengthening comprehensive cancer centres, implementing multidisciplinary care, enhancing education for oncology professionals, and expanding outpatient and day-care services to improve continuity of care and patient monitoring. Such system-level interventions may also have indirect but important implications for QoL by enabling earlier identification of patient needs and more timely supportive interventions.

In this context, our findings highlight the importance of structured follow-up care and the role of oncology nursing in addressing persistent physical and psychosocial burdens in breast cancer survivors. Although Slovakia has made progress in oncological care, challenges remain in integrating preventive, diagnostic and follow-up services into a fully coordinated system. Addressing these gaps is particularly relevant given the complexity of modern cancer treatment, which places increasing demands on specialized healthcare professionals, including oncology nurses. Strengthening education, clinical competencies and workforce capacity is therefore essential, as emphasized by international frameworks and professional oncology organizations, which highlight the critical role of nursing in improving patient outcomes [56].

Examples from high-resource settings, such as Norway [57], illustrate how integrated care models, active patient involvement and accessible communication channels, such as dedicated oncology support lines (e.g., “onco-nurse” hotlines), or digital monitoring tools and mobile applications, can support continuous symptom reporting and timely intervention [58]. In this context, oncology nurses play a central role in coordinating care, facilitating patient–provider communication, and ensuring timely responses to emerging clinical and psychosocial needs. These approaches may not only contribute to improved clinical outcomes but also to better long-term QoL.

Overall, our study confirms that breast cancer survivors experience persistent impairments across multiple QoL domains, driven by both physical and psychosocial factors. These findings highlight the need for tailored, long-term follow-up care that systematically integrates QoL assessment into routine clinical practice. Importantly, they reinforce the importance of oncology nurses in survivorship care, particularly in delivering individualized, patient-centred interventions and supporting patients across the physical, psychological and social dimensions of recovery. Strengthening oncology nursing involvement may therefore represent a key component in improving long-term QoL outcomes, particularly in healthcare systems where structured oncology nursing roles and survivorship care pathways are still evolving, such as Slovakia.

## 5. Conclusions

This study provides the first comprehensive assessment of QoL using the EORTC QLQ-C30 and BR23 questionnaires in Slovak breast cancer patients following treatment. The findings indicate that breast cancer is associated with a multidimensional impact on QoL, with the greatest impairments observed in social and role functioning, while physical, emotional and cognitive domains were relatively preserved. The most prominent symptoms included insomnia, fatigue, pain and upper limb complications, which reflects the persistent burden of both physical and psychosocial challenges. Differences in QoL were also observed in relation to treatment modalities and sociodemographic factors; however, these findings should be interpreted as associations and may be influenced by potential confounding factors.

The results highlight the importance of systematic QoL assessment in routine follow-up care and suggest that targeted supportive care interventions addressing both physical and psychosocial domains may be beneficial. Multidisciplinary supportive approaches, including rehabilitation, psychological support and patient education, are essential components of comprehensive survivorship care and may contribute to improved long-term outcomes.

Finally, this study highlights the need for ongoing research on long-term QoL outcomes in Slovak breast cancer patients and emphasizes the importance of integrating Slovak oncology centres into international QoL research networks to support improvements in care and survivorship within this specific healthcare context. Overall, these findings provide clinically relevant insights into QoL patterns in Slovak breast cancer patients, although the importance of cautious interpretation must be emphasized given the cross-sectional design of the study.

## Figures and Tables

**Table 1:** Cronbach’s alpha coefficients for EORTC QLQ-C30 scales.

Scale	Cronbach’s Alpha
Physical functioning	0.89
Role functioning	0.82
Emotional functioning	0.79
Cognitive functioning	0.66
Social functioning	0.86
Fatigue	0.88
Nausea & vomiting	0.44
Pain	0.93

**Table 2:** Profile of EORTC QLQ-C30 module and scale scores in patients.

	N	Mean	SD	min	max
*^a^* Global health status/QoL scale	244	51.67	15.24	25	83.33
*^a^* FS Physical functioning	244	67.33	19.90	26.67	100
*^a^* FS Role functioning	240	54.17	22.57	16.67	100
*^a^* FS Emotional functioning	242	74.58	21.09	33.33	100
*^a^* FS Cognitive functioning	244	81.67	12.97	66.67	100
*^a^* FS Social functioning	234	49.17	29.47	0	100
*^b^* SS Fatigue	244	36.11	23.57	0	100
*^b^* SS Nausea & vomiting	238	8.33	12.52	0	33.33
*^b^* SS Pain	242	32.50	23.56	0	66.67
*^c^* Dyspnoea	242	33.33	23.87	0	66.67
*^c^* Sleep disturbance	244	40	30.31	0	100
*^c^* Appetite loss	238	23.33	21.62	0	66.67
*^c^* Constipation	234	20	24.81	0	66.67
*^c^* Diarrhoea	236	8.33	18.10	0	66.67
*^c^* Financial impact	215	16.67	22.65	0	66.67

Legend: QoL—quality of life, N—number of respondents, SD—standard deviation. *^a^* For functional scales (FS)—higher scores indicate better functioning, *^b^* for symptom scales (SS) and *^c^* individual items—higher scores indicate worse functioning. Note: The number of observations (N) varies across variables due to item-level missing or invalid responses. Only valid responses were included in accordance with the EORTC scoring guidelines.

**Table 3:** Cronbach’s alpha coefficients for EORTC QLQ-BR23 scales.

Scale	Cronbach’s Alpha
Body image	0.90
Sexual functioning	0.81
Systemic side effects	0.56
Arm symptoms	0.82
Breast symptoms	0.91

**Table 4:** Evaluation of the QoL of patients in terms of functional status and symptoms (EORTC QLQ-BR23).

	N	Mean	SD	min	max
*^a^* FS Body image	244	71.67	24.66	0	100
*^a^* FS Future perspective	240	30.00	25.93	0	66.7
*^a^* FS Sexual functioning	210	13.32	16.52	0	33.3
*^a^* FS Sexual enjoyment	186	46.15	16.91	33.3	66.7
*^b^* SS Systemic side effects	232	23.33	13.11	9.5	47.6
*^b^* SS Hair loss	240	27.78	23.92	0	66.7
*^b^* SS Arm symptoms	242	23.89	23.14	0	77.8
*^b^* SS Breast symptoms	238	18.75	20.82	0	66.7

Legend: QoL—quality of life, N—number of respondents, SD—standard deviation. *^a^* For functional scales (FS)—higher scores indicate better functioning, *^b^* for symptom scales (SS)—higher scores indicate worse functioning. Note: The number of observations (N) varies across variables due to missing or invalid responses at the item level. Only valid responses were included in accordance with the EORTC scoring guidelines.

**Table 5:** Comparison of the QoL of breast cancer patients in terms of individual functional scales.

Functional Scales of QoL	Description				Friedman Analysis of Variance	
	MR	M	Mdn	SD	χ^2^(7)	*p*
Cognitive functioning	6.65	81.67	83.33	12.97	173.368	<0.001
Emotional functioning	6.13	74.58	79.17	21.09		
Body image	5.93	71.67	75.00	24.66		
Physical functioning	5.43	67.33	70.00	19.90		
Role functioning	4.23	54.17	50.00	22.57		
Social functioning	3.85	49.17	50.00	29.47		
Future perspective	2.48	30.00	33.33	25.93		
Sexual functioning	1.33	13.32	0.00	16.52		

QoL—quality of life, M—arithmetic mean, MR—mean rank, Mdn—median, SD—standard deviation, χ^2^(7)—Friedman’s analysis of variance (degrees of freedom in brackets), *p*—statistical significance.

**Table 6:** Comparison of the QoL of breast cancer patients in terms of the severity of individual groups of symptoms.

Symptoms	Description				Friedman Analysis of Variance	
	MR	M	Mdn	SD	χ^2^(11)	*p*
Fatigue	8.73	36.11	33.33	23.57	103.311	<0.001
Sleep disturbance	8.43	40.00	33.33	31.31		
Dyspnoea	8.40	33.33	33.33	23.87		
Pain	7.85	32.50	33.33	23.56		
Appetite loss	6.85	23.33	33.33	21.62		
Systemic side effects	6.70	23.33	19.05	13.11		
Constipation	6.13	20.00	0.00	24.81		
Arm symptoms	6.10	23.89	22.22	23.14		
Breast symptoms	5.40	18.75	12.5	20.82		
Financial impact	5.05	16.67	0.00	22.65		
Nausea & vomiting	4.28	8.33	0.0	12.52		
Diarrhoea	4.10	8.33	0.0	18.10		

QoL—quality of life, M—arithmetic mean, MR—mean rank, Mdn—median, SD—standard deviation, χ^2^(11)—Friedman’s analysis of variance (degrees of freedom in brackets), *p*—statistical significance.

**Table 7:** Comparison of QoL in terms of individual functional scales in breast cancer patients depending on whether they underwent chemotherapy.

Functional Scales	Chemotherapy						Mann–Whitney U Test	
	No (N = 118)			Yes (N = 126)				
	AM	Mdn	SD	AM	Mdn	SD	U	*p*
Physical functioning	65.46	73.33	18.59	69.63	66.67	21.72	170.0	0.443
Role functioning	45.46	50.00	16.41	64.82	66.67	24.85	108.0	0.011
Emotional functioning	76.52	83.33	21.15	72.22	75.00	21.39	172.0	0.473
Cognitive functioning	83.33	83.33	12.60	79.63	83.33	13.47	166.0	0.353
Social functioning	48.49	50.00	28.60	50.00	66.67	31.31	188.0	0.782
Body image	49.24	50.00	14.30	54.63	58.33	16.23	150.0	0.185
Future perspective	77.27	83.33	16.70	64.82	66.67	30.99	174.0	0.486
Sexual functioning	27.27	33.33	24.42	33.33	33.33	28.01	174.0	0.442
Sexual enjoyment	15.14	0.00	16.97	11.10	0.00	16.15	14.00	0.299
Global health status/QoL scale	50.00	50.00	17.85	39.98	33.30	14.94	162.0	0.321

Legend: QoL—quality of life, N—number of respondents, AM—group mean, SD—standard deviation, Mdn—median, U—Mann–Whitney U test, *p*—statistical significance.

**Table 8:** Comparison of symptom severity in breast cancer patients according to whether they received chemotherapy.

Symptoms	Chemotherapy						Mann–Whitney U Test	
	No (N = 118)			Yes (N = 126)				
	AM	Mdn	SD	AM	Mdn	SD	U	*p*
Fatigue	41.41	33.33	23.30	29.63	33.33	22.87	144.0	0.137
Nausea & vomiting	10.61	0.00	13.16	5.56	0.00	11.43	154.0	0.157
Pain	39.39	33.33	20.92	24.07	33.33	24.40	130.0	0.043
Dyspnoea	30.30	33.33	22.79	37.04	33.33	25.28	168.0	0.375
Sleep disturbance	51.52	33.33	30.39	25.93	33.33	26.95	110.0	0.012
Appetite loss	21.21	33.33	16.41	25.93	33.33	26.95	186.0	0.717
Constipation	15.15	0.00	26.68	25.93	33.33	21.56	138.0	0.069
Diarrhoea	6.06	0.00	13.16	11.11	0.00	22.87	186.0	0.639
Financial impact	15.15	0.00	26.68	18.52	33.33	17.04	162.0	0.260
Systemic side effects	23.38	19.05	13.70	23.28	23.81	12.75	196.0	0.956
Hair loss	0.00	0.00	0.00	41.67	33.33	15.43	0.00	0.003
Arm symptoms	29.29	22.22	28.80	17.28	22.22	10.93	160.0	0.283
Breast symptoms	25.00	16.67	23.85	11.11	0.00	13.41	124.0	0.039

Legend: N—number of respondents, AM—group mean, SD—standard deviation, Mdn—median, U—Mann–Whitney U test, *p*—statistical significance.

**Table 9:** Comparison of QoL in terms of individual functional scales in breast cancer patients depending on whether they underwent radiotherapy.

Functional Scales	Radiotherapy						Mann–Whitney U Test	
	No (N = 91)			Yes (N = 153)				
	AM	Mdn	SD	AM	Mdn	SD	U	*p*
Physical functioning	70.00	70.00	15.40	65.56	70.00	22.56	172.0	0.578
Role functioning	54.17	50.00	20.64	54.17	58.33	24.20	188.0	0.909
Emotional functioning	70.83	79.17	23.96	77.08	75.00	19.07	164.0	0.433
Cognitive functioning	83.33	83.33	14.91	80.56	83.33	11.70	172.0	0.556
Social functioning	33.33	25.00	24.34	59.72	58.33	28.20	96.0	0.007
Body image	50.00	54.17	14.91	52.78	50.00	15.67	96.0	0.007
Future perspective	57.29	62.50	30.56	81.25	83.33	13.52	100.0	0.007
Sexual functioning	16.67	0.00	24.34	38.89	33.33	23.40	160.0	0.298
Sexual enjoyment	16.65	16.65	17.20	11.10	0.00	16.04	13.00	0.225
Global health status/QoL scale	53.34	66.70	18.29	41.65	33.30	15.46	182.0	0.780

Legend: QoL—quality of life, N—number of respondents, AM—group mean, SD—standard deviation, Mdn—median, U—Mann–Whitney U test, *p*—statistical significance.

**Table 10:** Comparison of symptom severity in breast cancer patients according to whether they received radiotherapy.

Symptom	Radiotherapy						Mann–Whitney U Test	
	No (N = 91)			Yes (N = 153)				
	AM	Mdn	SD	AM	Mdn	SD	U	*p*
Fatigue	31.94	33.33	17.63	38.89	38.89	26.82	160.0	0.371
Nausea & vomiting	4.17	0.00	7.45	11.11	0.00	14.47	148.0	0.151
Pain	29.17	33.33	26.87	34.72	33.33	21.38	166.0	0.452
Dyspnoea	41.67	33.33	22.77	27.78	33.33	23.40	132.0	0.071
Sleep disturbance	37.50	33.33	26.87	41.67	33.33	34.40	186.0	0.862
Appetite loss	20.83	33.33	16.67	25.00	33.33	24.57	180.0	0.713
Constipation	25.00	16.67	28.55	16.67	0.00	21.98	164.0	0.389
Diarrhoea	4.17	0.00	11.39	11.11	0.00	21.23	166.0	0.303
Financial impact	12.50	0.00	16.67	19.44	0.00	25.85	172.0	0.526
Systemic side effects	22.62	21.43	9.76	23.81	16.67	15.12	182.0	0.779
Hair loss	33.33	33.33	29.81	22.22	33.33	17.21	14.00	0.484
Arm symptoms	23.61	22.22	23.96	24.07	22.22	23.09	188.0	0.909
Breast symptoms	10.42	4.17	12.73	24.31	20.83	23.43	124.0	0.044

Legend: N—number of respondents, AM—group mean, SD—standard deviation, Mdn—median, U—Mann–Whitney U test, *p*—statistical significance.

**Table 11:** Multiple linear regression analysis of factors associated with overall health and QoL in breast cancer patients.

Predictors	Global Health Status/QoL Scale				
	B	SE	β	t	*p*
Constant	41.913	19.69		2.129	0.041
Age	0.0540	0.162	0.058	0.336	0.739
Place of residence *^a^*	−13.402	5.397	−0.443	−2.483	<0.001
Education level	−5.2140	5.242	−0.193	−3.995	0.018
Chemotherapy *^b^*	3.1500	4.541	0.104	0.694	0.493
Radiotherapy *^c^*	1.0340	4.825	0.034	0.214	0.832
MODEL	Adj.R^2^ = 0.253		F(5.34) = 3.640		*p* = 0.010

*^a^* reference group are female patients living in the city (urban), *^b^* reference group are female patients who did not undergo chemotherapy, *^c^* reference group are female patients who did not undergo radiotherapy. QoL—quality of life, B—unstandardized regression coefficient, SE—standard error of B, β—standardized regression coefficient, *p*—statistical significance, Adj.R^2^—indicator of the degree of explained variance of the dependent variable of the model, F—predictive power of the regression model.

## Data Availability

Data supporting the reported results can be provided on demand in Excel form. Personal data are not available due to GDPR EU regulations; however, anonymized extraction of clinical data from the Slovakian patient’s journal systems can be provided on demand.

## Public Involvement Statement

Patients were involved in this research as study participants. They completed standardized QoL questionnaires (EORTC QLQ-C30 and QLQ-BR23) independently and provided data reflecting their experiences after breast cancer treatment. Patients or the public were not involved in the design of the study, development of research questions, outcome measures, data analysis, interpretation of results, manuscript preparation, or reporting or dissemination of this research.

## Guidelines and Standards Statement

This manuscript was drafted in accordance with the STROBE (Strengthening the Reporting of Observational Studies in Epidemiology) Statement guidelines for reporting cross-sectional observational studies [59].
